# The Clinical Significance of Vitamin D in Systemic Lupus Erythematosus: A Systematic Review

**DOI:** 10.1371/journal.pone.0055275

**Published:** 2013-01-30

**Authors:** Rajalingham Sakthiswary, Azman Ali Raymond

**Affiliations:** Department of Medicine, Universiti Kebangsaan Malaysia Medical Centre, Cheras, Kuala Lumpur, Malaysia; University of Leuven, Rega Institute, Belgium

## Abstract

**Background:**

Vitamin D deficiency is more prevalent among SLE patients than the general population. Over the past decade, many studies across the globe have been carried out to investigate the role of vitamin D in SLE from various clinical angles. Therefore, the aim of this systematic review is to summarise and evaluate the evidence from the published literature; focusing on the clinical significance of vitamin D in SLE.

**Methods:**

The following databases were searched: MEDLINE, Scopus, Web of Knowledge and CINAHL, using the terms “lupus”, “systemic lupus erythematosus”, “SLE and “vitamin D”. We included only adult human studies published in the English language between 2000 and 2012.The reference lists of included studies were thoroughly reviewed in search for other relevant studies.

**Results:**

A total of 22 studies met the selection criteria. The majority of the studies were observational (95.5%) and cross sectional (90.9%). Out of the 15 studies which looked into the association between vitamin D and SLE disease activity, 10 studies (including the 3 largest studies in this series) revealed a statistically significant inverse relationship. For disease damage, on the other hand, 5 out of 6 studies failed to demonstrate any association with vitamin D levels. Cardiovascular risk factors such as insulin resistance, hypertension and hypercholesterolaemia were related to vitamin D deficiency, according to 3 of the studies.

**Conclusion:**

There is convincing evidence to support the association between vitamin D levels and SLE disease activity. There is paucity of data in other clinical aspects to make firm conclusions.

## Introduction

The link between vitamin D and systemic lupus erythematosus (SLE) was first described in 1995 [1]. The discovery of vitamin D receptor expression by cells of the immune system has spurred more research on the immunomodulatory properties of vitamin D over the past decade. Both the innate and adaptive immune systems have a wide array of cells such as macrophages, dendritic cells, T cells, and B cells which express vitamin D receptors that may respond to the biologically active form of vitamin D (1,25-dihydroxyvitamin D) [2]. Several studies across the globe have reported that vitamin D deficiency is more prevalent among SLE patients than the general population [3]. A possible explanation for this is the sun avoidance by SLE patients, which is an established trigger of lupus flares.

To date, there are over a hundred studies on SLE and vitamin D. The investigators of these studies have tried to establish the prevalence of vitamin D deficiency and its significance in various clinical aspects such as disease activity, disease damage and laboratory parameters. A question which is yet to be answered is whether or not vitamin D deficiency truly alters the course and prognosis of SLE. The answer to this question has important clinical implications, as it may offer potential therapeutic possibilities with vitamin D supplementation. Therefore, the aim of this systematic review is to summarise and evaluate the evidence from published literature focusing on the clinical significance of vitamin D in SLE.

## Methodology

### Search Strategy

We used the terms “lupus”, “systemic lupus erythematosus”, “SLE”, “vitamin D” and ‘SLE and vitamin D” to search the following databases: MEDLINE, Scopus, Web of Knowledge and CINAHL. Furthermore, the references of all retrieved articles were reviewed for relevant citations.

### Inclusion Criteria

All adult human cohort and case-control studies written in English, which investigated the role and effects of vitamin D in SLE published between the years 2000 and 2012 were included.

### Exclusion Criteria

Studies in other languages apart from English, case reports, case series, animal studies letters to the editor and review articles were excluded. Regarding the justification for excluding paediatric studies; apart from the age factor, paediatric SLE runs a more aggressive clinical course than adult SLE with higher rates of organ involvement and the disease tends to be more severe at presentation [4], [5]. Besides, studies on vitamin D receptor gene polymorphisms were not selected. Most of the aforementioned studies lacked emphasis on and were not powered to investigate the correlation between measured vitamin D levels (25[OH]D) and its clinical significance [6], [7], [8]. Stringent selection criteria were applied in order to achieve a high level of homogeneity in the studies included in this systematic review.

### Screening of Articles for Eligibility

**Figure 1:**
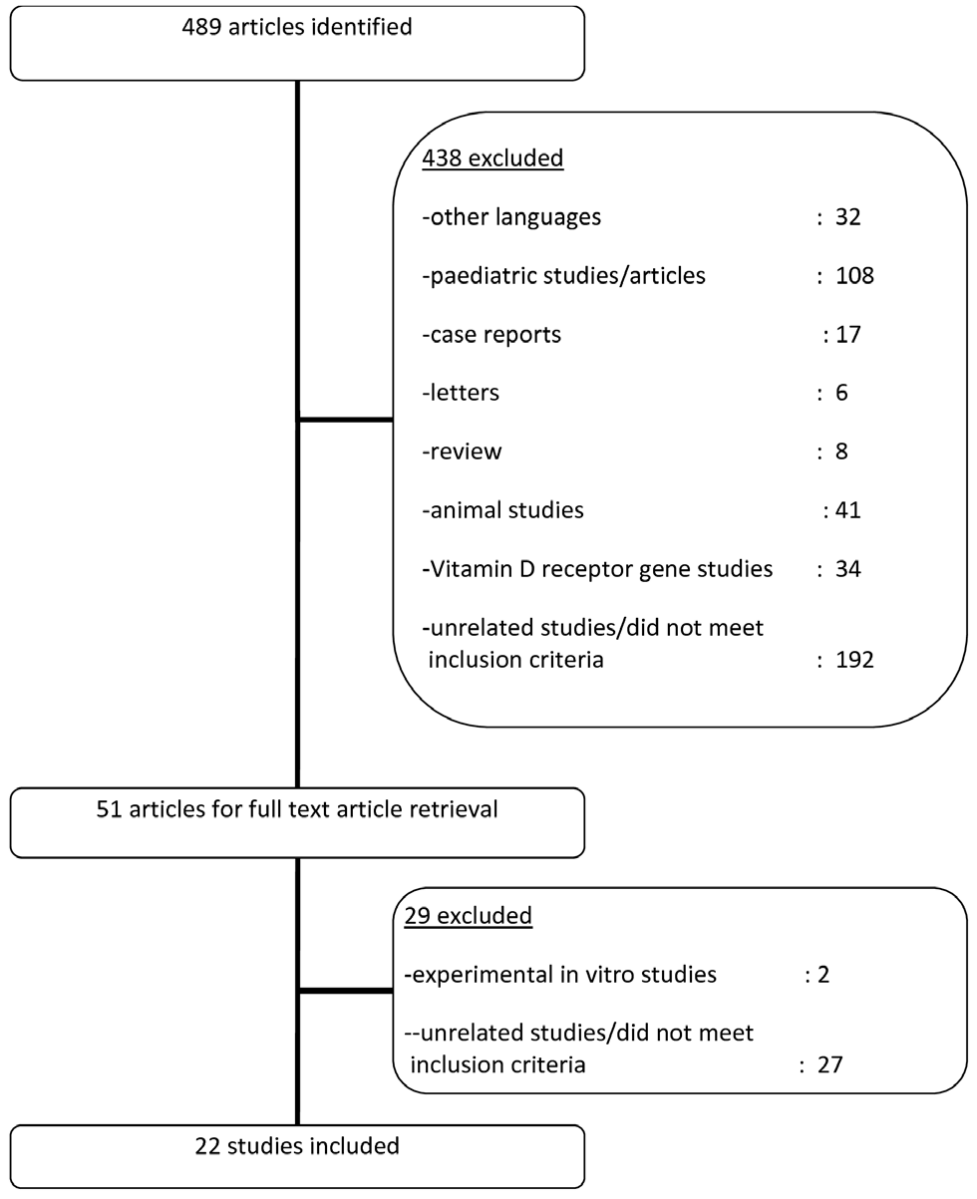
The algorithm for selection of studies in this systematic review.

Retrieved articles were screened for eligibility based on titles and abstracts and were subsequently classified as ‘include’, ‘possible’ and ‘exclude’ categories. The ‘include’ and ‘possible’ categories comprised studies reporting (1) measured vitamin D levels and/or vitamin D supplementation, and (2) disease activity, disease damage, laboratory parameters and/or organ involvement in SLE. In the ‘possible’ category, there were uncertainties concerning the study design, sample population or objectives. For articles falling into the ‘include’ and ‘possible’ categories, where available, the full texts were obtained and assessed. Both authors participated in the selection of articles and only articles which were agreed upon by both were finally included in the review. Throughout the selection process, both authors reached consensus in every instance. Figure 1 summarises the algorithm followed in the selection of studies.

### Data Extraction

The following data were extracted from the selected studies: study design; country; year; sample size; disease activity and damage scoring systems; organ involvement and findings. The relevant and particularly, significant statistical values were recorded (odds ratios [OR], p values, rho [r]).

## Results

A total of 22 studies met the eligibility criteria. The vast majority of the studies were observational (95.5%) and cross-sectional (90.9%). Eight case-control studies and 14 cohort studies were included. The number of studies originating from each continent is as follows: 8 from America, 6 from Europe, 6 from Asia, 1 from Australia and 1 from Africa. Study sample sizes varied from 37 to 378 subjects. All studies used 25-hydroxyvitamin D to determine the vitamin D level. Table 1 highlights the findings of the selected studies.

**Table 1:** Summary of the selected studies.

Ref.	Year	Country	Study design	Study population	Findings/Conclusions	Statistical findings
[31]	2006	United States	Cross sectional Case-control	123 recently diagnosed SLE 240 controls	Lower vitamin D levels is associated with a. presence of renal disease b. photosensitivity.	OR 13.3, p<0.01 OR 12.9, p<0.01
[9]	2008	Spain	Cross sectional Cohort	92 SLE	No relation was seen between vitamin D and disease duration, SLEDAI, SLICC-ACR or VAS indexes.	
[27]	2008	United States	Cross sectional Cohort	37 female SLE	Vitamin D deficiency was associated with a. lower global assessment scores, However, levels of dsDNA were higher in the group with levels of vitamin D that were greater than 47.7 nmol/L	p< or = 0.003p = 0.0069
[32]	2009	Canada	Cross sectional Cohort	124 SLE	Vitamin D levels showed no correlation with bone mineral density	p = 0.26
[15]	2009	Brazil	Cross sectional Case-control	36 SLE 26 controls	Vitamin D level was associated witha. SLEDAI,b. osteocalcinc. bone-specific alkaline phosphatase.	r = −0.65, p<0.001)r = 0.35r = −0.17,
[10]	2009	United States	Cross sectionalCohort	181 female SLE	Lower vitamin D levels were significantly associated with highera. diastolic blood pressureb. low-density lipoprotein cholesterol,c. lipoprotein(a)d.fibrinogen levelse. self-reported hypertensionf. diabetes mellitusg. SLEDAIh. SLICC.With further adjustment for BMI, these associations were no longer significant.	p = 0.034p = 0.013p = 0.024p = 0.000p = 0.016,OR 0.68p = 0.032, OR 0.49p = 0.001p = 0.003
[11]	2010	Spain	Prospective cohort,those with low baseline vitamin D levels were supplemented with oral vitamin D(3)	80 SLE	Inverse significant correlations between vitamin D levels and the VAS(fatigue)Changes in vitamin D levels correlated with changes in the VAS in patients with baseline vitamin D levels <30 ng/mlNo significant correlationsbetween the vitamin D levels and:a. SLEDAIb. SDI	p = 0.001p = 0.017p = 0.87p = 0.63
[16]	2010	Israel	Cross sectionalCohort study	378 SLE(European and Israeli patients)	A significant negative correlation between the serum concentration of vitamin D and the SLEDAI-2K and ECLAM scales	r = −0.12,p = 0.018.
[17]	2011	Egypt	Cross sectionalCase-control	60 SLE60 controls	Serum vitamin D levels were lower witha. increased SLEDAI score,b. frequency of photosensitivity	OR: 2.72,p = 0.002OR: 3.6,p<0.01
[18]	2011	Iran	Cross sectionalCohort study	40 SLE	Serum vitamin D concentration was inversely correlated with the BILAG index score.Vitamin D deficiency was associated witha. higher concentrations of liver enzymes,b. lower serum albumin and hemoglobin concentrationsc. higher titers of antibodies to double-stranded DNA (ds-DNA).	r = −0.486,p = 0.001p<0.05p<0.05p<0.001
[25]	2011	United States	Cross sectionalCase-control	32 SLE32 controls	Vitamin D deficiency was associated witha. higher B cell activationb. higher serum IFNalpha activity	p = 0.009p = 0.02
[12]	2011	Korea	Cross sectionalCase-control	104 SLE49 controls	The serum vitamin D levels, were positively correlated only witha. hemoglobinb. serum complement 3but not witha. SLEDAIb. SLICC	beta = 0.256, p = 0.018beta = 0.365, p = 0.002beta = −0.04,p = 0.742beta = −0.052,p = 0.62
[19]	2011	Hungary	Cross sectionalCohort	177 SLE	Reduced vitamin D levels were associated with :a. pericarditisb. neuropsychiatric diseasesc. deep vein thrombosisd. higher SLEDAI scoree. higher anti-double-stranded (ds)DNA autoantibody concentrations,f. higher anti-Smith antigen (anti-Sm) concentrationsg. lower C4 levelsh. higher immunoglobulin (Ig)G concentration	p = 0.013p = 0.010p = 0.014p = 0.038p = 0.021p<0.001p = 0.027p = 0.034
[28]	2012	Australia	Cross sectionalCase-control	24 SLE21 controls	Fatigue was not related to vitamin D status	
[13]	2012	Spain	Cross sectionalCohort study	73 SLE	No correlation between vitamin D deficiency anda. SLEDAI scoreb. SLICC/ACR score	p = 0.310p = 0.820
[14]	2012	Hong Kong	Cross sectionalCohort study	290 SLE	Vitamin D correlated inversely and significantly witha. clinical SLE activityb. anti-C1qc. anti-dsDNA titers,d. but not with complement levels or damage scores.	r = −0.26;p<0.001r = −0.14;p = 0.020r = −0.13;p = 0.020
[20]	2012	Hong Kong	Cross sectionalCohort	290 SLE	Levels of vitamin D correlated inversely witha. PGA,b. total SLEDAI scoresvitamin D deficiency had significantly highera. total/high-density lipoprotein(HDL) cholesterol ratiob. prevalence of antiphospholipid syndromeNo association could be demonstrated between vitamin D level and atherosclerosis	beta −0.20;p = 0.003beta −0.19;p = 0.003p = 0.02p = 0.007
[21]	2012	Malaysia	ProspectiveCohort	38premenopausal SLE	There was a significant negative correlation between SLEDAI scores and vitamin D levels.	p = 0.033
[26]	2012	Poland	Cross sectionalCase-control	49 SLE.49 controls	Vitamin D deficiency was associated witha. renal diseaseb. leucopeniac. lower serum concentrations of IL-23)	p = 0.006p = 0.047p = 0.037
[23]	2012	Brazil	Cross sectionalCase control	78 SLE64 controls	No statistically significant association was observed between vitamin D deficiency and the following:a. disease activity (SLEDAI >6)b. fatiguec. anti-DNA	p = 0.971p = 0.808p = 0.435
[22]	2012	UnitedKingdom	Cross sectionalCohort	75 SLE	Patients with vitamin D deficiency had highera. BMIb. insulin resistance.c. SLEDAI-2KAortic stiffness was inversely associated with serum vitamin D independently of BMI, CVD risk factors and serum insulin.There was no association between vitamin D and carotid plaque and intima media thickness.	p = 0.014p = 0.023p = 0.031beta = −0.0217p = 0.010
[29]	2012	UnitedStates	Cross sectionalCohort	51 SLE	vitamin D levels inversely correlated with age-adjusted total plaque area.	r = –0.33,p = 0.018

### Vitamin D and its Association with SLE Disease Activity and Damage

The disease activity was assessed in most studies with validated scoring systems such as SLEDAI (Systemic Lupus Erythematosus Disease Activity Index), BILAG (British Isles Lupus Activity Group), and ECLAM (European Consensus of Lupus Activity Measurement). In 6 of the studies, the disease damage was investigated with the Systemic Lupus International Collaborating Clinics Damage Index (SDI) [9], [10], [11], [12], [13], [14]. Across the studies, the correlation between vitamin D levels and SLE disease activity has been rather inconsistent. Out of the 15 studies which looked into this aspect, two third of the studies (10/15) reported a significant inverse association between vitamin D and the measured disease activity [10], [14], [15], [16], [17], [18], [19], [20], [21], [22]. There were no significant methodological variations between the 10 studies showing an inverse relationship between vitamin D and the disease activity and the 5 studies that did not show such relationship. The outcome measurement used in all the 5 studies was SLEDAI [9], [11], [12], [13], [23]. Similarly, 90% (9/10) of the studies which demonstrated a significant inverse relationship used SLEDAI. Of note, 3 out of the 5 negative studies in this regard were Spanish [9], [11], [13], and 2 of them were by the same authors [9], [11].The damage scores, on the other hand, in 5 out of 6 studies failed to demonstrate a significant association with vitamin D levels [9], [11], [12], [13], [14].

In clinical practice, anti double stranded (ds) DNA and complement levels are simple, yet useful tools to monitor SLE disease activity. High titres of antidsDNA and low complement levels are associated with lupus flares [24].The majority of studies showed a significant inverse relationship between vitamin D levels and the former [14], [18], [19] and a direct relationship with the latter [12], [19]. Along this line, Ritterhouse et al. [25] found that that vitamin D deficiency was associated with significantly higher B cell activation and interferon alpha activity. Bogaczewicz et al. [26] had conflicting results in this regard with their finding of vitamin D deficiency being associated with lower concentrations of interleukin 23.

### Vitamin D and VAS (Visual Analogue Score)

The VAS was used to evaluate the patients’ global assessment [9], [20], [27] and level of fatigue [11], [23], [28]. The findings of these studies showed no consistent pattern with respect to the relationship between the VAS and vitamin D levels. Only half of the studies found significant inverse correlation between these parameters [11], [20], [27]. The remaining studies found no significant association.

### Vitamin D and Cardiovascular Risk Factors

There were 4 studies which investigated the relationship between vitamin D deficiency and cardiovascular risk factors such as hypercholesterolaemia, insulin resistance/diabetes mellitus and hypertension [10], [20], [22], [29]. Wu et al. [10] found a significant association with insulin resistance which was consistent with the results of the study by Reynolds et al. [22]. Mok et al. [20] demonstrated a higher total/high density lipoprotein (HDL) cholesterol ratio in vitamin D deficient subjects supporting the findings of Wu et al. [10] of higher low density lipoprotein (LDL) cholesterol with lower vitamin D levels. Reynolds et al. [22] and Ravenell et al. [29] had conflicting results on the correlation with carotid plaque. Ravenell et al. [29] discovered that vitamin D levels inversely correlated with age-adjusted total plaque area while Reynolds et al. [22] found the opposite. The latter, however demonstrated a significant increase in aortic stiffness with reducing levels of vitamin D.

## Discussion

The results of this systematic review indicate that there is substantial evidence to convince us of the association between vitamin D levels and SLE disease activity. However, one may argue that a few of the studies refute this finding and cast doubts on the aforementioned link. It is noteworthy that the 3 largest studies with sample sizes of 378,290 and 181 subjects, revealed strong inverse correlations between vitamin D levels and SLEDAI scores with p values of 0.018,<0.001 and 0.001, respectively [10], [14], [16]. Mok et al. [14] concluded that vitamin D deficiency is a marker of SLE disease activity with comparable specificity to anti-C1q. The conflicting results of the few studies could be due to the diverse study populations, methodological variations and the power of several studies [11], [13], [23] was probably too low to achieve statistical significance. While the results of these observational studies are helpful for generating hypotheses concerning the effects of vitamin D on the clinical course of SLE, it is unwise to make causal inferences from these studies.

Lupus disease activity predicts the risk of subsequent organ damage [30]. Intriguingly, the majority of studies consistently demonstrated that vitamin D level is not associated with organ damage [9], [11], [12], [13], [14]. The authors are unable to provide any valid evidence-based explanation for this observation. This finding limits or perhaps, deters the use of vitamin D levels as a prognostication tool. Kamen et al. (4) and Bogaczewicz et al. (22) found an association between vitamin D levels and renal disease. Similarly, Reynolds et al. (24) and Ravenell et al. (25) pointed out a significant relationship with the cardiovascular system. These findings need to be tested rigorously by larger prospective studies to make firm conclusions. As these studies are too few in number, there is still profound lack of evidence to support the role of vitamin D in the management of the individual organ system. Nevertheless, this is an area worthy of further research due to the biologic plausibility of a link between vitamin D deficiency and especially, cardiovascular and renal disease in SLE.

Our systematic review has limitations. We did not include articles in other languages which may have had valuable information or additional evidence related to this topic. It is reasonable to assume that some studies with negative or null results were simply not published; a well recognised publication bias. The cross sectional study design used in the majority of these studies does not give us a clear picture as to whether vitamin D deficiency confers a poorer outcome of SLE in the long term. Future research on vitamin D in SLE will hopefully address more practical concerns and provide answers to the following questions : the most appropriate phase of SLE to assess vitamin D (ie, at the time of diagnosis or while in remission); the cutoff value of ‘normal’ versus ‘insufficient’ vitamin D levels in lupus patients as compared to the general population; potential confounding factors such as medications, age, body size, geographic location, ethnicity, sun protective behaviours; and genetic variation in the metabolism of vitamin D.

Much emphasis has been placed on vitamin D in SLE in recent years. Apart from its significant association with disease activity, based on the evidence highlighted in this systematic review, it is premature and would be fallacious to make any definitive claims for or against the role of vitamin D in other clinical aspects.
